# BNIP3-dependent mitophagy role in cisplatin resistance

**DOI:** 10.1080/27694127.2022.2131210

**Published:** 2022-10-06

**Authors:** Veronica Cocetta, Caterina Vianello, Marta Giacomello, Monica Montopoli

**Affiliations:** a Department of Pharmaceutical and Pharmacological Sciences, University of Padova, Largo E. Meneghetti 2, 35131, Padova, Italy; b Department of Biology, University of Padova, Via Ugo Bassi 58B, 35131, Padova, Italy; c Department of Biomedical Sciences, Via Ugo Bassi 58B, 35131, Padova, Italy; d Veneto Institute of Molecular Medicine, Via Orus 2, 35129, Padova, Italy

**Keywords:** autophagy inhibitors, cancer, chemotherapy resistance, mitochondria, mitophagy

Chemotherapy resistance is one of the major limits to cancer treatment and overall patient survival: it represents a compelling challenge for oncology researchers. Cisplatin exerts its therapeutic efficacy by interfering with DNA replication but also by interacting with other nucleophilic species within cells, including side chains of mitochondrial proteins and nucleotides. Mitochondria are key signaling hubs that fundamentally contribute to cancer development and progression. Despite mitochondria being well-known for their contribution in cellular bioenergetics, little is known about their role in chemoresistance.

In a recent paper by Vianello *et al*., the involvement of mitochondria in resistance to the commonly used chemotherapeutic drug cisplatin (CDDP) has been explored in two cancer cell lines, 2008 (ovarian carcinoma) and U2OS (osteosarcoma), and their CDDP-resistant isogenic derivative clones (respectively, C13 and U2OS-PT) [1]. The authors uncovered for the first time that BNIP3 (BCL2 interacting protein 3), a key receptor for selective mitochondrial autophagic machinery, is highly expressed in cisplatin-resistant cells, suggesting its pivotal role in chemotherapy response. How the crosstalk between autophagy/mitophagy and pro-survival/pro-death signaling influences tumor development and cancer resistance to chemotherapy is still under debate.

The mitochondria-dependent cell death process is counterbalanced by their selective degradation via autophagy (mitophagy) as a pro-survival defense mechanism against cell stressors. Autophagy is essential for maintaining cellular homeostasis and is rapidly triggered by different stimuli such as nutrient availability alterations, oxidative stress and hypoxia. Specifically, mitophagy is the selective removal of damaged mitochondria by autophagic machinery, and mitochondrial fission is synchronized with mitophagy, linking this dynamic event to the mitochondrial degradation process.

It was observed that in cisplatin-resistant cancer cells (C13 and U2OS-PT), mitochondria morphology is different from that observed in their sensitive counterpart, appearing more fragmented and less interconnected [1]. Building on this, the hypothesis that mitochondrial dynamics (that is, broadly speaking, all the processes that control their shape, number and motility) can play a role in the onset of resistance appeared intriguing. Electron micrography and morphometric analysis indicated a reduced perimeter of mitochondria in resistant cells as compared to 2008 and U2OS CDDP-sensitive cells. The mitochondrial number, size, and position within the cytoplasm are regulated by mitochondrial morphological changes influenced by fusion and fission processes. Analysis of key factors involved in mitochondrial dynamics revealed higher levels of pro-fission proteins (*FIS1* mRNA and MFF protein in C13; *FIS1* and *DNM1L/DRP1* mRNA and MFF protein in U2OS-Pt) and lower levels of the pro-fusion protein OPA1 in resistant cells. Conversely, higher levels of the mitochondrial outer membrane fusion protein MFN2 have been detected in resistant cells. MFN2 also contributes to tether the endoplasmic reticulum (ER) to mitochondria, allowing Ca^2+^ transfer from the ER to mitochondria and maintaining control of pro-survival/pro-death pathways. Further analysis of electron micrographs and a FRET-based assay confirm an increased proximity among the two organelles in resistant cells. As mentioned above the mitophagy receptor BNIP3 is overexpressed in CDDP-resistant cells. Taking into account these results, the authors supposed that mitophagy can influence the degree of drug resistance and the overall tumor response to chemotherapy by providing a rapid turnover of damaged mitochondria, thus promoting cell survival. The BNIP3 overexpression observed in *in vitro* cell models has been confirmed also in ovarian cancer patient samples. Bioinformatics analysis of RNA-seq datasets generated by the TCGA consortium confirm an increased expression of *BNIP3* in patients with lower cancer progression-free survival intervals. To corroborate the hypothesis that mitophagy contributes to chemotherapy response, it has been demonstrated *in vitro* that BNIP3 knockdown can restore the sensitivity to cisplatin in resistant cells by reducing the mitophagy flux. As further confirmation, a pharmacological approach based on PIK-III and SAR405, two specific inhibitors for PIK3C3/Vps34, a lipid kinase controlling autophagosome formation, revealed that inhibition of autophagy increases CDDP efficacy in resistant cancer cells.

This recent work shows for the first time the implication of BNIP3 in the phenomenon of cisplatin resistance and the possibility to target the autophagic/mitophagic machinery as a strategy to improve cisplatin efficacy in resistant tumors. Considering the double-edged sword that autophagy represents in cancer progression, further studies are guaranteed: nevertheless, this work represents a step forward in the understanding of platinum resistance mechanisms, providing new hits for the development of future therapeutic strategies.
